# Evidence of Direct Toxicological Effects of Scorpion Venom on Central Nervous System in Tunisian Children

**DOI:** 10.1155/2018/8304375

**Published:** 2018-10-23

**Authors:** Mabrouk Bahloul, Basma Souissi, Olfa Turki, Mariem Dlela, Khaireddine Ben Mahfoudh, Mounir Bouaziz

**Affiliations:** ^1^Department of Intensive Care, Habib Bourguiba University Hospital, Sfax, Tunisia; ^2^Department of Radiology, Habib Bourguiba University Hospital, Sfax, Tunisia

## Abstract

**Background:**

Severe scorpion envenomation can lead to severe neurological manifestations, which are an indicator of the severity of the scorpion sting. The direct action of scorpion venom on the central nervous system can explain partly these neurological disorders.

**Methods and Findings:**

We report a case of severe scorpion envenomation in 16-month-old boy with no pathological history admitted in ICU for severe scorpion envenomation. The result of cerebral MRI agrees with the hypothesis of direct action of scorpion venom on the central nervous system. Patient had improved; however, he has kept as neurological sequelae language disorders and blindness. The boy was discharged 21 days after ICU admission.

**Conclusion:**

Our observation confirms that severe scorpion envenomation can be complicated by severe neurological manifestations. Although one case report is not enough to conclude such important hypothesis regarding the direct effect of scorpion venom on central nervous system (especially that the age of patient is more than one year), our case agrees with this hypothesis.

## 1. Introduction

Severe scorpion envenomation can lead to severe neurological manifestations, which are an indicator of the severity of the scorpion sting. The direct action of scorpion venom on the central nervous system can explain partly these neurological disorders in particular in children with immature hematoencephalic barrier [1]. However this mechanism was in our knowledge never confirmed in humans. We report a case of severe scorpion envenomation in 16-month-old boy with no pathological history admitted in ICU for severe scorpion envenomation. The result of cerebral MRI agrees with the hypothesis of direct action of scorpion venom on the central nervous system.

## 2. Observation

A 16-month-old boy with no pathological history was brought to the emergency department for severe scorpion envenomation (*Androctonus australis species*). Clinical examination on admission showed systemic manifestations with nausea, vomiting, agitation, and sweating. Moreover, this child exhibited signs of vital distress with pulmonary edema (pulse oximetry [SpO2] was at 70% on air room), a circulatory failure (with cardiogenic shock), and neurological failure (coma with Glasgow coma scale score at 5/15). The patient had undergone intubation and mechanical ventilation. Moreover, the patient received scorpion antivenom. Cardiogenic shock and pulmonary edema were treated by Dobutamine.

On ICU admission, cardiac function was explored by echocardiography showing a low left ventricular ejection fraction (LVEF), at 20% under Dobutamine.  Table 1 details clinical characteristics of our patient.

During ICU stay, a control echocardiography performed 7 days after the initial study showed complete recovery, with an LVEF of 0.65.

**Figure 1:**
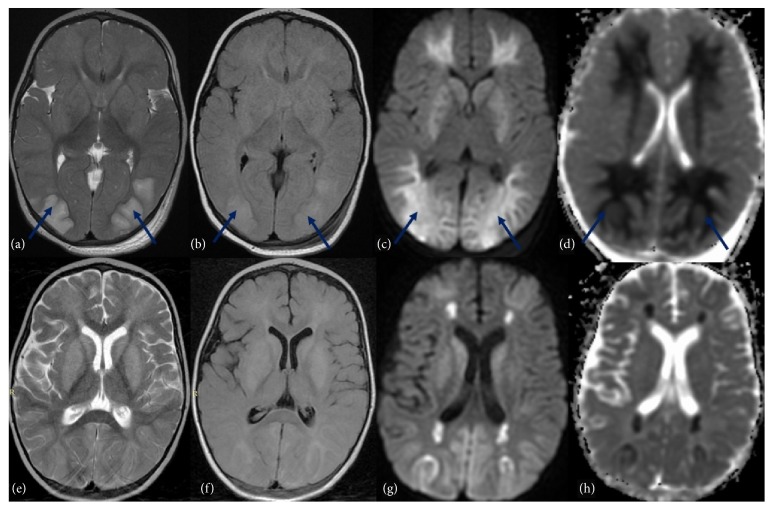
MRI scans: (a, e) T2-weighted; (b,f) Fluid Attenuated Inversion Recovery (FLAIR); (c,g) diffusion-weighted imaging; (d,h) apparent diffusion coefficient (ADC) map. The first MRI imaging (a,b,c,d) showed bilateral and symmetrical hyperintensity (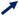) inT2, FLAIR and diffusion of the occipital and frontal lobes, cerebellar hemispheres, periventricular white matter, and basal ganglia with low ADC related to cytotoxic edema. After 9 days, control MRI (e, f, g, h) highlighted progressive volume loss of cerebrum with decreased hypersignal and increased ADC in cerebellar hemispheres, fontal and occipital lobes. Hypersignal persists in T2, FLAIR, and diffusion in periventricular white matter with ADC restriction and in basal ganglia without ADC restriction.

A brain MRI scan with sagittal T1 spin echo (SE), Axial T1SE/T2 SE/FLAIR and diffusion, and apparent diffusion coefficient (ADC) map was performed (within 24 hours after ICU admission), showing a cytotoxic cerebral edema (Figure 1) with bilateral and symmetrical hyperintensity inT2, FLAIR, and diffusion of the occipital lobes, cerebellar hemispheres, and periventricular white matter with low ADC related to cytotoxic edema. Control MRI performed 9 days later showed a great improvement of cerebral damage with progressive volume loss of cerebrum with decreased hypersignal and increased ADC in cerebellar hemispheres, fontal and occipital lobes. Hypersignal persists in T2, FLAIR, and diffusion in periventricular white matter with ADC restriction and in basal ganglia without ADC restriction (Figure 1).

Respiratory and circulatory parameters were improved under the treatments previously detailed. However, the patient has kept as neurological sequelae language disorders and blindness. The boy was discharged 21 days after ICU admission.

## 3. Discussion

The result of MRI performed on ICU admission agrees with the hypothesis of the possibility of direct action of scorpion venom on the central nervous system in children.

Severe scorpion envenomation can be complicated by severe neurological manifestations, which are an indicator of the severity of the scorpion sting. These signs are observed in more than 50% of hospitalized patients [1–3]. These manifestations were variable, ranging from simple hyperthermia to severe neurological manifestations with coma and/or convulsions [1–5]. These neurological manifestations have been explained by three mechanisms. The first is hypertensive encephalopathy secondary to the massive catecholamine's discharge [1–3]. The second is the brain hypoxia due to the deficiency in oxygen transport secondary to the pulmonary edema and cardiogenic shock observed in severe scorpion envenomation. The last is the direct action of scorpion venom on the central nervous system. This hypothesis was advanced by Ismail and coll [4], who postulated the possibility of the venom to cross the hematoencephalic barrier in immature children, like the passage in immature mammals of the scorpion venom [4].** In fact, t**he correlation between the young age and severity of clinical manifestations after scorpion envenomation was well established [1–3]. This correlation between the age and the severity may be explained by the fact that, for the same quantity of venom injected, the serum levels of venom would be higher in children than in adults, leading to the possibility of the venom to cross the hematoencephalic barrier in immature children, like our cases. In fact, there is a good correlation between serum venom concentrations and severity of neurological manifestations [1, 3, 5–7].

Our patient had developed pulmonary edema and cardiogenic shock. However, brain damage observed in the cerebral MRI cannot be explained only by these abnormalities. In fact, as cytotoxic oedema represents the redistribution of water from extracellular to intracellular compartments, there is an appropriate decrease in diffusion, identified as low signal on apparent diffusion coefficient (ADC) [8]. This cytotoxic edema can be explained by a direct effect of the venom on the central nervous system. This can explain the severity of neurological manifestation observed on ICU admission and the neurological sequelae observed in our patient.

## 4. Conclusion

Scorpion stings occur on every continent except Antarctica. The correlation between young age and severity of clinical manifestations is well established. Cardiorespiratory manifestations, mainly cardiogenic shock and pulmonary edema, are the leading causes of death after scorpion envenomation. Neurological manifestations were often observed in severe scorpion-envenomed patients and they correlated with poor outcome. Severe neurological complications can be observed following severe scorpion envenomation. These neurological manifestations have been explained by many mechanisms. Although one case report is not enough to conclude such important hypothesis regarding the direct effect of scorpion venom on central nervous system (especially that the age of patient is more than one year), our case agrees with this hypothesis. More studies are needed on this subject.

## Figures and Tables

**Table 1:** Clinical parameters recorded on ICU admission.

**Parameters**	**Results**
Age (Years)	16 months

Sex	Male

Severity	Grade III (with cardiogenic shock and pulmonary edema) and severe neurological manifestations

Respiratory rate (breaths/min)	28/min

Heart rate	180/min

Glasgow coma scale score	5/15

Shock	Yes

Use of mechanical ventilation	Yes

pH	6.95

Bicarbonate level (mmol/l)	6

PaO2/FiO2 ratio	70

Echocardiography result	low left ventricular ejection fraction (LVEF), at 20%

Scorpion antivenom (GAMMA-SCORP®: bivalent scorpion antivenom, Institut Pasteur, Tunis, Tunisia)	Yes (1ml/kg)
